# Paradoxical Herniation following Decompressive Craniectomy in the Subacute Setting

**DOI:** 10.1155/2016/2090384

**Published:** 2016-06-30

**Authors:** Alex P. Michael, Jose Espinosa

**Affiliations:** Department of Surgery, Division of Neurosurgery, Southern Illinois University School of Medicine, P.O. Box 19645, Springfield, IL 62794-9645, USA

## Abstract

Decompressive craniectomy is reserved for extreme cases of intracranial hypertension. An uncommon complication known as paradoxical herniation has been documented within weeks to months following surgery. Here we present a unique case within days of surgery. Since standard medical treatment for intracranial hypertension will exacerbate paradoxical herniation, any abrupt neurological changes following decompressive craniectomy should be carefully investigated. Immediate treatment for paradoxical herniation is placement of the patient in the supine position with adequate hydration. Cranioplasty is the ultimate treatment option.

## 1. Background

Decompressive craniectomy is a radical surgical intervention reserved for extreme cases of intracranial hypertension after failed attempts of maximum medical therapy. By removing the bone flap, the craniectomy exposes the once closed cranial vault to atmospheric pressure and allows the brain to herniate outwards instead of toward the vital subcortical structures. Currently, one of the few indications for decompressive craniectomy is malignant middle cerebral artery (MCA) infarction characterized by intracranial hypertension and rapid neurological deterioration [1]. An uncommon complication of hemicraniectomy is “sinking skin flap” (SSF) syndrome also known as syndrome of the trephined. Occasionally the sunken skin flap may progress to “paradoxical herniation” of the intracranial components over the course of several weeks to months [2]. Here, we present a unique instance of paradoxical herniation within days of decompression without any inciting event other than positional change of the patient.

## 2. Clinical Presentation

A 58-year-old Caucasian male with a past medical history of hypertension, hyperlipidemia, and previous stroke resulting in right-sided weakness was admitted to a general surgical floor in May 05, 2015, to recover from an uncomplicated L5-S1 hemilaminectomy. He was taking aspirin 325 mg and Plavix 75 mg qday and both were stopped prior to his surgical procedure. On postoperative day number two, the patient had an acute change in mental status along with left hemiparesis. CT of the head revealed no acute process. He was seen by a neurologist via telemedicine and suspected to have a right MCA stroke. He was intubated and sedated due to clinical deterioration and transferred to the regional stroke center for further management.

**Figure 1:**
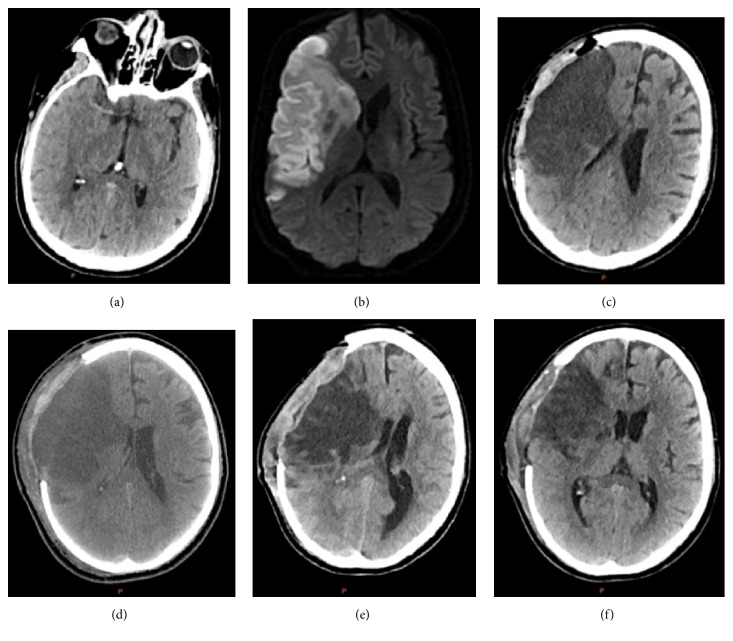
(a) Original CT angiogram revealing a dense right MCA sign. (b) Initial MRI with diffusion weighted sequencing. (c) Postcraniectomy CT. (d) Postop day #4 s/p hemicraniectomy. (e) Sunken flap CT, POD #8. (f) Head CT postop day #12.

After transport, a CTA of head and neck (Figure 1(a)) revealed a right MCA infarct with dense right MCA sign indicating an occlusion in the M2 segment. Bilateral internal carotid artery occlusions were also noted to begin near the carotid origins and extend to the skull base. Due to the patient's recent surgery, tPA administration was contraindicated. Transthoracic echocardiograph revealed a left ventricular ejection fraction of 64% without intracardiac thrombus, patent foramen ovale, or wall motion abnormality. A CT perfusion was performed and reviewed by the interventional neuroradiologist. The patient was not deemed to be a candidate for intervention as there was little penumbra. Subsequent MRI with DWI sequence confirmed a large right-sided MCA territory infarction (Figure 1(b)).

Upon neurosurgical examination, the patient was difficult to arouse and did not open eyes to voice or pain. Pupils were 3 mm, equal, round, and sluggishly reactive to light. A right gaze preference was present. The patient moved extremities to painful stimuli, however, with gross left sided weakness when compared to the right. He was able to follow commands to give thumbs up and show two fingers though he remained intubated and nonverbal.

He was restarted on aspirin 325 mg and lovenox for DVT prophylaxis and started on 3% hypertonic saline to maintain a serum sodium level between 145 and 155. Plavix was stopped due to high risk of hemorrhagic conversion of the large territory infarction. On day two following the acute infarct, the patient's mental status progressed to coma and was no longer following commands. He was taken by neurosurgery for a decompressive right hemicraniectomy (Figure 1(c)). On postoperative day number 1, the patient was again following commands to show two fingers. Significant swelling was noted at the side of craniectomy but the skin flap was soft. Pupils were equal and reactive at 3 mm bilaterally. The patient continued an unremarkable postoperative recovery in the neurocritical care unit (Figure 1(d)).

On postoperative day 8, the nursing staff noted an unreactive 4 mm right pupil. Left pupil was 3 mm and reactive and the patient was unresponsive to verbal or painful stimuli. On visual inspection, the skin flap overlying the craniectomy was sunken. A stat CT was performed and revealed a 16 mm right to left shift with associated mass effect upon the brainstem (Figure 1(e)).

The patient continued to exhibit a sunken flap within hours of placement in the upright position. During these episodes, he became obtunded, developed dysconjugate gaze, and at one occurrence developed a fully dilated right pupil. These symptoms resolved promptly with Trendelenburg positioning and hydration. Cranioplasty consisting of replacement of the bone flap was completed approximately three months from the original craniectomy and was followed by complete resolution of positional deteriorations (Figure 1(f)).

## 3. Discussion

Alterations of intracranial pressure and cerebrospinal fluid dynamics following hemicraniectomy predispose patients to the well described syndrome of the trephined characterized by headaches, mood changes, dizziness, and seizures [3]. Occasionally, atmospheric pressure and gravitational forces are great enough to overwhelm the intracranial contents and lead to paradoxical herniation toward the subcortical structures.

Previously documented instances of paradoxical herniation following decompressive craniectomy occur exclusively over weeks to months following surgical intervention [2]. We have presented a unique case of severe paradoxical herniation following decompression occurring less than 10 days after surgical intervention. Case studies thus far have revealed numerous instances of paradoxical herniation due to altered CSF hydrodynamics following external ventriculostomy, lumbar puncture, or ventriculoperitoneal shunting [4–6]. These procedures further lower intracranial pressure states relative to extracranial pressures. In the previously presented case, no such CSF alteration had taken place. Importantly, no incidental durotomy or CSF leak was encountered during the initial lumbar spine surgery. Retrospective analysis found that those patients with sinking skin flap syndrome had significantly smaller surface craniectomy, tended to be older in age, and had a larger infarct volume. One hypothesis has been that atrophy of the infarcted tissue leads to a decrease in the intracranial volume and subsequently a decrease in intracranial pressure over time [7]. It is highly unlikely that adequate time passed for our patient to have any appreciable brain atrophy to alter pressure dynamics.

Paradoxical herniation cannot be reversed with traditional treatments for herniation syndromes, as these will effectively exacerbate paradoxical herniation. Paradoxical measures are needed to acutely raise the intracranial pressures and counter the external atmospheric pressure such as Trendelenburg position, hydration, and discontinuation of both hyperosmolar therapy and CSF drainage. The definitive treatment appears to be cranioplasty to return the system to its original “closed box” state. Increased cerebral blood flow after cranioplasty has been reported suggesting that a decrease in flow may be a contributing factor for development of SSF syndrome [8]. Currently, the ideal timing for cranioplasty is unknown though most are performed at 2-3 months following decompressive craniectomy, once cerebral edema has dissipated. Up until that time, patients may continue to have deterioration as in the case of our patient. SSF syndrome and paradoxical herniation may delay neurological recovery in the interim.

## 4. Conclusion

Although paradoxical herniation has been documented in the literature following decompressive craniectomy, it is typically encountered weeks to months after the initial surgery or following changes to CSF hydrodynamics. Here we have described a more expeditious onset of this phenomenon without a known inciting event. In the acute and subacute setting of malignant cerebral edema, clinicians may assume that neurological deteriorations are due to intracranial hypertension and thus treat them accordingly with traditional medical interventions such as hyperosmolar therapy and hyperventilation. It has been shown that these common measures would exacerbate paradoxical herniation and could lead to irreparable damage by the time the true pathology is discovered. Therefore, following any abrupt neurological changes after decompressive craniectomy, it is important to immediately inspect the contour and tenseness of the cranial flap prior to initiation of medical therapy and to ultimately assess the subcortical structures with CT imaging. Irrespective of the timing of onset of paradoxical herniation, the most emergent treatment seems to be placement of the patient in the supine position with adequate hydration. Cranioplasty continues to be considered the ultimate treatment option.
